# Resveratrol regulates mitochondrial reactive oxygen species homeostasis through Sirt3 signaling pathway in human vascular endothelial cells

**DOI:** 10.1038/cddis.2014.530

**Published:** 2014-12-18

**Authors:** X Zhou, M Chen, X Zeng, J Yang, H Deng, L Yi, M-t Mi

**Affiliations:** 1 Research Center for Nutrition and Food Safety, Chongqing Key Laboratory of Nutrition and Food Safety, Institute of Military Preventive Medicine, Third Military Medical University, Chongqing Medical Nutrition Research Center, Chongqing 400038, PR China

## Abstract

Mitochondrial reactive oxygen species (mtROS) homeostasis plays an essential role in preventing oxidative injury in endothelial cells, an initial step in atherogenesis. Resveratrol (RSV) possesses a variety of cardioprotective activities, however, little is known regarding the effects of RSV on mtROS homeostasis in endothelial cells. Sirt3 is a mitochondrial deacetylase, which plays a key role in mitochondrial bioenergetics and is closely associated with oxidative stress. The goal of the study is to investigate whether RSV could attenuate oxidative injury in endothelial cells via mtROS homeostasis regulation through Sirt3 signaling pathway. We found that pretreatment with RSV suppressed tert-butyl hydroperoxide (t-BHP)-induced oxidative damage in human umbilical vein endothelial cells (HUVECs) by increasing cell viability, inhibiting cell apoptosis, repressing collapse of mitochondrial membrane potential and decreasing mtROS generation. Moreover, the enzymatic activities of isocitrate dehydrogenase 2 (IDH2), glutathione peroxidase (GSH-Px) and manganese superoxide dismutase (SOD2) as well as deacetylation of SOD2 were increased by RSV pretreatment, suggesting RSV notably enhanced mtROS scavenging in t-BHP-induced endothelial cells. Meanwhile, RSV remarkably reduced mtROS generation by promoting Sirt3 enrichment within the mitochondria and subsequent upregulation of forkhead box O3A (FoxO3A)-mediated mitochondria-encoded gene expression of ATP6, CO1, Cytb, ND2 and ND5, thereby leading to increased complex I activity and ATP synthesis. Furthermore, RSV activated the expressions of phosphorylated adenosine monophosphate-activated protein kinase (p-AMPK), peroxisome proliferator-activated receptor gamma coactivator-1*α* (PGC-1*α*) and Sirt3, as well as estrogen-related receptor-*α* (ERR*α*)-dependent Sirt3 mRNA transcription, which were abolished in the presence of AMPK inhibitor and *AMPK*, *PGC-1α* or *Sirt3* siRNA transfection, indicating the effects of RSV on mtROS homeostasis regulation were dependent on AMPK-PGC-1*α*-ERR*α*-Sirt3 signaling pathway. Our findings indicated a novel mechanism that RSV-attenuated oxidative injury in endothelial cells through the regulation of mtROS homeostasis, which, in part, was mediated through the activation of the Sirt3 signaling pathway.

Atherosclerotic cardiovascular disease remains the main cause of death in the United States. Numerous studies have demonstrated that atherosclerosis (AS) is the result of a prolonged and excessive response to injury in the vascular wall, which begins with oxidative damage in the endothelium, characterized by endothelial dysfunction.^1^ Oxidative stress, which can be defined as an imbalance between the production of endogenous reactive oxygen species (ROS) and the presence of antioxidant molecules, is one of the most important mechanisms contributing to endothelial dysfunction.^2^ It has been recognized that the mitochondria are the main source of ROS intracellularly and cardiovascular risk factors are associated with excessive mitochondrial ROS (mROS) production that promotes oxidative stress and inflammation, and decreases nitric oxide bioavailability. Moreover, vascular endothelial cells are highly glycolytic and the mitochondrial content is relatively low in comparison with other cell types with higher energy requirements.^3, 4, 5^ Thus, it was indicated that the mitochondria in endothelial cells are to play a limited role in energy production but, sense the local environment the endothelial cells face and orchestrate the cellular homeostasis and function by virtue of its ROS-producing capacity.^6^ In the normal condition, the mtROS participate in critical signaling pathways to mediate adaptive responses and regulate diverse biological functions. However, excess mtROS production can directly induce endothelial dysfunction through stimulating the production of proinflammatory cytokines. Thus, development of drugs, which maintain mtROS homeostasis could be crucial for preventing endothelial dysfunction.

Resveratrol (3, 4′, 5-trihydroxystilbene, RSV) is a polyphenolic compound found mainly in the skins of grapes, and berry fruits (cranberry, strawberry, mulberry, etc.), and in peanuts and red wine. Accumulating evidence suggests that RSV is a highly pleiotropic molecule that modulates abundant targets and thus influences numerous biochemical and molecular functions.^7^ Epidemiological studies indicate that the Mediterranean diet, which is rich in RSV, is associated with a reduced risk of carotid AS.^8^ Evidence from clinical trials indicated that RSV may protect against AS by improving gene expression in the vascular endothelium, and could be considered as a preventive agent.^9^ We previously found that RSV could penetrate into vascular endothelial cells and attenuate endothelial inflammation by inducing autophagy through the cyclic adenosine monophosphate (cAMP) signaling pathway.^10, 11^ Nonetheless, little information is available regarding the effects of RSV on mtROS homeostasis regulation in endothelial cells.

Recent studies suggested that silent mating-type information regulation 2 homolog 3 (Sirt3), plays a key role in regulating mtROS homeostasis.^12^ Sirt3 is targeted to the mitochondrial matrix where it orchestrates mitochondrial oxidative metabolism, such as the TCA cycle and oxidative phosphorylation (OXPHOS), through deacetylation of a variety of substrates. Intriguingly, Sirt3 also governs the level of mtROS by deacetylation of the major mitochondrial antioxidant enzymes, including manganese superoxide dismutase (SOD2), isocitrate dehydrogenase 2 (IDH2) and glutathione peroxidase (GSH-Px), as well as the components for mtROS generation in the electron transport chain (ETC) such as complex I and complex III.^13, 14^ Moreover, it has been reported that Sirt3 functions as a downstream target of peroxisome proliferator-activated receptor (PPAR*γ*) coactivator-1*α* (PGC-1*α*), which is directly regulated by adenosine monophosphate-activated protein kinase (AMPK), thereby stimulating deacetylation of mitochondrial enzymes involved in mtROS homeostasis and activation of forkhead box O3A (FoxO3A)-mediated mitochondrial DNA (mtDNA) transcription to attenuate oxidative damage.^12, 15, 16^ Recent work highlights the critical importance of Sirt3 in the regulation of energetic metabolism by RSV treatment in the liver through stimulating complex I activity.^17^ Thus, we hypothesized that RSV could attenuate oxidative injury in endothelial cells via mtROS homeostasis regulation through Sirt3 signaling pathway. As expected, our results indicated, for the first time, that RSV-attenuated oxidized injury in endothelial cells induced by oxidative damage through regulation of mtROS homeostasis, which, in part, was mediated through the activation of the Sirt3 signaling pathway. These results provide new evidence regarding the vascular protective effects of RSV and may also open new avenues for finding new drugs that can be applied to endothelial protection and AS prevention.

## Results

### RSV inhibited t-BHP-induced endothelial injury in human umbilical vein endothelial cells

**Figure 1:**
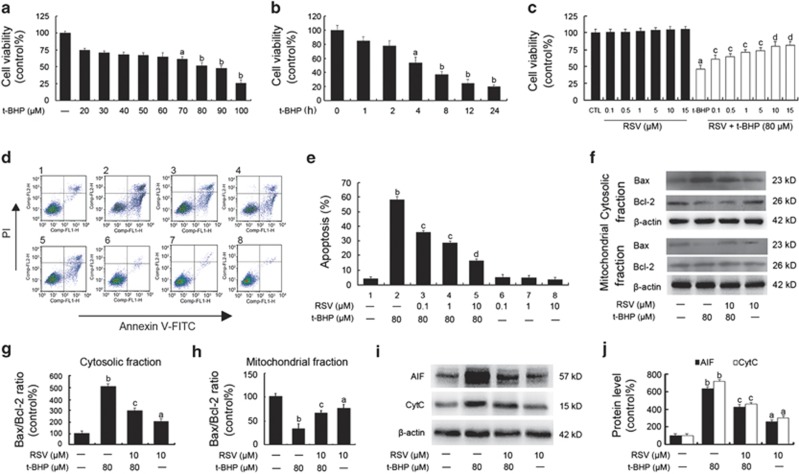
RSV inhibited t-BHP-induced injury in HUVECs. (**a** and **b**) Cells were treated with t-BHP with different concentrations (20, 30, 40, 50, 60, 70, 80, 90 and 100 *μ*M) for 4 h (**a**) or for different time intervals (1, 2, 4, 8, 12 and 24 h; **b**), respectively. Cell viability was determined using the CCK-8 assay and data are expressed as percentage of the control. (**c**) Confluent cells were pretreated for 2 h with various concentrations of RSV (0.1, 0.5, 1, 5, 10 and 15 *μ*M). After removing the supernatants, cells were incubated with fresh medium in the presence or absence of t-BHP (80 *μ*M) for an additional 4 h. Cell viability was determined using the CCK-8 assay. (**d**) Representative images of flow cytometric analysis by annexin V-FITC/PI dual staining. The bottom right quadrant represents annexin V-FITC-stained cells (early-phase apoptotic cells) and the top right quadrant represents PI- and annexin V-FITC-dual-stained cells (late-phase apoptotic/necrotic cells). (**e**) Apoptotic cells are represented as the percentage of annexin-V single-positive plus annexin-V/PI double-positive cells. (**f**) After the indicated treatments, the cells were harvested and lysed to detect the cytoplasmic and mitochondrial levels of Bax and Bcl-2 by western blot analysis. (**g**) The bar graphs show the ratio of Bax/Bcl-2 in the cytosolic fraction in endothelial cells. (**h**) The bar graphs show the ratio of Bax/Bcl-2 in the mitochondrial fraction in endothelial cells. (**i**) The expressions of AIF and CytC in the cytosolic fraction of endothelial cells were detected. (**j**) The bar graphs show the quantification of the indicated proteins. All results are representative of three independent experiments and values are presented as means±S.E.M. (*n*=3). ^a^*P*<0.05, ^b^*P*<0.01 *versus* the control group; ^c^*P*<0.05, ^d^*P*<0.01 *versus* the t-BHP-treated group

As shown in Figures 1a and b, tert-butyl hydroperoxide (t-BHP) decreased cell viability in a dose-dependent (Figure 1a) and time-dependent (Figure 1b) manner in human umbilical vein endothelial cells (HUVECs), respectively. The cell viability was reduced to 52±2.16% when treated with t-BHP (80 *μ*M) for 4 h compared to the control group. However, RSV pretreatment significantly attenuated the detrimental effect of t-BHP on cell viability and apoptosis in HUVECs (*P*<0.05; Figures 1c–e). And RSV (0.1, 1, 10 and 15 *μ*M) alone had no notable effects on cell viability and apoptosis in HUVECs (*P*>0.05; Figures 1c–e). Moreover, the expressions of apoptosis-related factors including apoptosis-inducing factor (AIF), cytochrome *c* (Cytc), Bcl-2-associated X protein (Bax) and B-cell lymphoma-2 (Bcl-2) were measured by flow cytometry. As shown in Figures 1f–h, the expression of Bax was decreased significantly in the mitochondrial fraction but increased significantly in the cytosolic fraction in HUVECs when treated with t-BHP, compared with the control. However, Bcl-2 decreased notably in the cytosolic fraction as a result of t-BHP treatment, while there seemed no significant changes of Bcl-2 expressions in the mitochondrial fractions. Consequently, the Bax/Bcl-2 ratio after t-BHP treatment decreased significantly in the mitochondrial fraction, but increased in the cytosolic fraction in HUVECs, while resveratrol pretreatment inhibited t-BHP-induced changes of Bax/Bcl-2 ratio both in cytosolic and mitochondrial fractions. Meanwhile, RSV pretreatment decreased the cytosolic expressions of AIF and Cytc in t-BHP-induced endothelial cells (Figures 1i and j). Taken together, these results suggest that RSV pretreatment remarkably attenuated t-BHP-induced oxidative injury in HUVECs.

### RSV attenuated t-BHP-induced collapse of mitochondrial membrane potential in HUVECs

**Figure 2:**
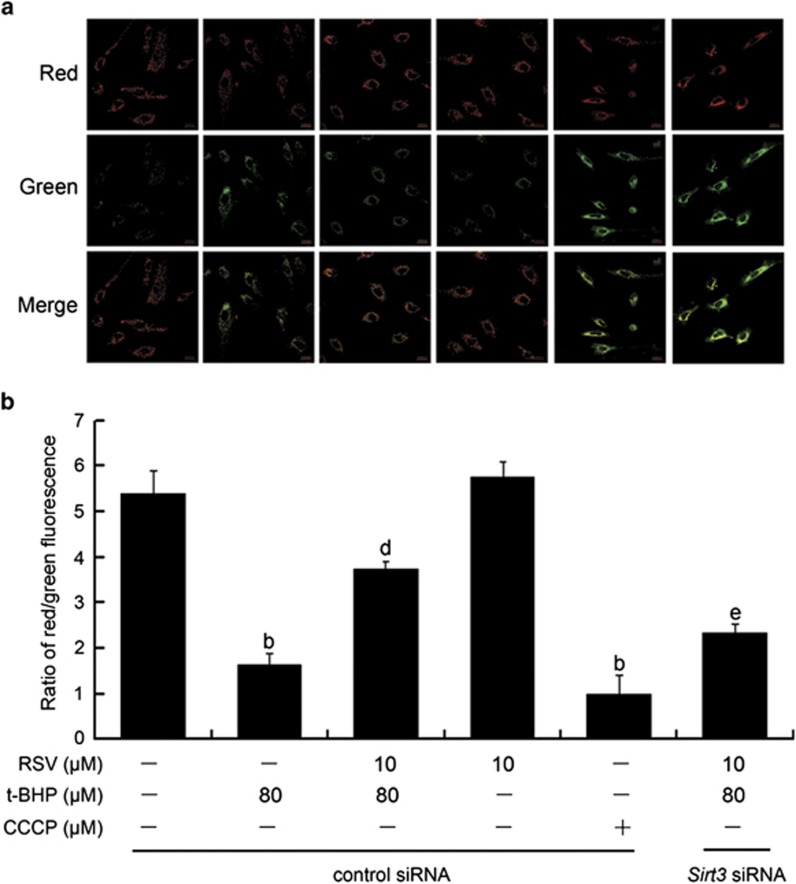
RSV suppressed t-BHP-induced collapse of mitochondrial membrane potential in HUVECs. Sirt3 was knocked down by *Sirt3* siRNA transfection as described in the Materials and Methods section. At 24-h post-transfection, cells were pretreated with RSV of 10 *μ*M for 2 h, washed, and then treated with or without t-BHP of 80 *μ*M for an additional 4 h. (**a**) Determination of Δ*ψ*m was carried out using CLSM. Red fluorescence was emitted by JC-1 aggregates in healthy mitochondria with polarized inner mitochondrial membranes, whereas green fluorescence was emitted by cytosolic JC-1 monomers, indicating Δ*ψ*m dissipation. Merged images indicate co-localization of JC-1 aggregates and monomers. (**b**) Δ*ψ*m in each group was calculated as the ratio of red to green fluorescence. All results are presented as mean±S.E.M. of at least three independent experiments. ^b^*P*<0.01 *versus* the control group; ^d^*P*<0.01 *versus* the t-BHP-treated group; ^e^*P*<0.05 *versus* RSV (10 *μ*M) and t-BHP (80 *μ*M) co-treated group with control siRNA transfection

The effect of RSV on t-BHP-induced mitochondrial membrane potential (Δ*ψ*m) in HUVECs was measured using JC-1 probe. JC-1 can selectively enter mitochondria and reversibly changes color as the Δ*ψ*m changes.^18^ As shown in Figures 2a and b, there was a significantly increased green fluorescence in cells exposed to t-BHP (80 *μ*M; *P*<0.05), suggesting that the mitochondrial membrane was depolarized. However, RSV (10 *μ*M) pretreatment repressed the changes of Δ*ψ*m induced by t-BHP, indicated by a reduction of green fluorescence and restoration of red fluorescence. Moreover, *Sirt3* siRNA transfection abolished the effects of RSV on t-BHP-induced changes of Δ*ψ*m in HUVECs. These results suggested that RSV remarkably suppressed t-BHP-induced collapse of mitochondrial membrane potential in a Sirt3-dependent manner in HUVECs.

### RSV attenuated t-BHP-induced mitochondrial dysfunction by inhibiting mtROS generation in HUVECs

**Figure 3:**
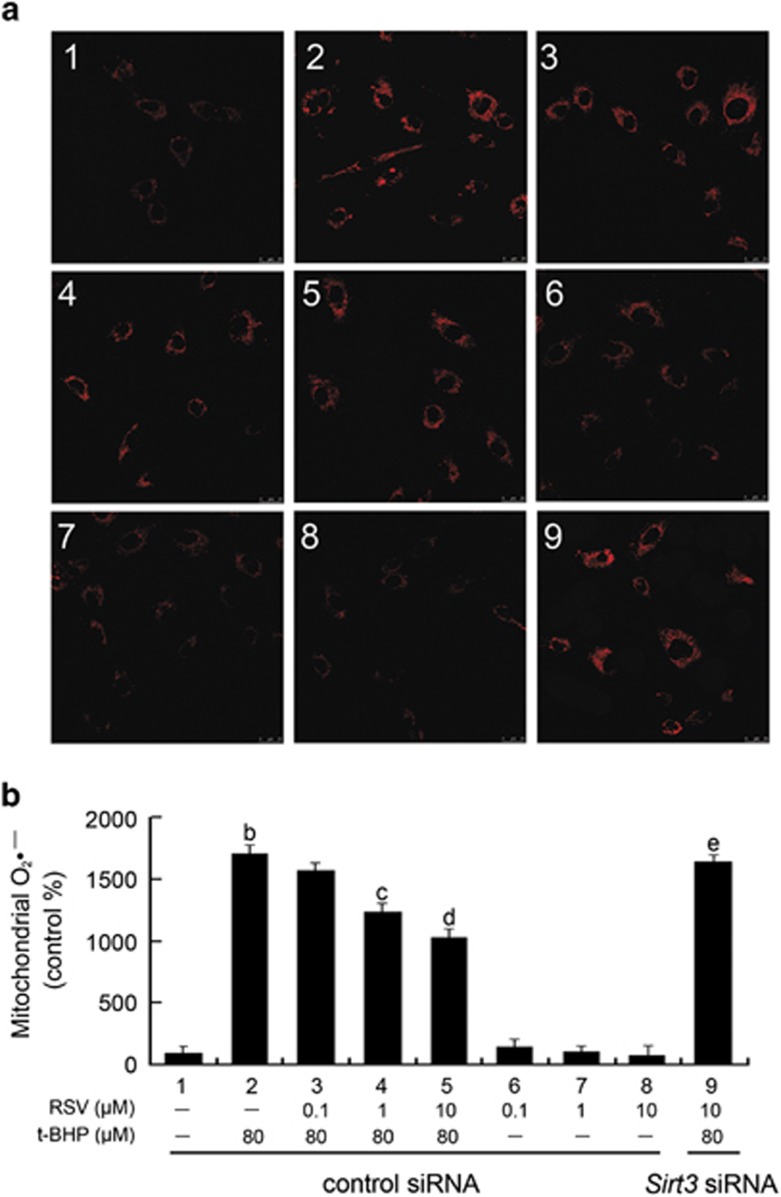
RSV attenuated t-BHP-induced mitochondrial dysfunction by inhibiting mtROS generation in HUVECs. Cells were transfected with *Sirt3* siRNA as described in the Materials and Methods section. At 24-h post-transfection, cells were pretreated with RSV (0.1, 1 and 10 *μ*M) for 2 h, washed, and then incubated with fresh medium in the presence or absence of t-BHP (80 *μ*M) for an additional 4 h. (**a**) The mitochondrial O_2_^•−^ levels were estimated using MitoSOX Red and the fluorescence values were read at an excitation wavelength of 510 nm and emission wavelength of 579 nm. (**b**) The bar charts show quantification of the mitochondrial O_2_^•−^ levels expressed as the fold change relative to the control group. All results are presented as means±S.E.M. of at least three independent experiments. ^b^*P*<0.01 *versus* the control group; ^c^*P*<0.05, ^d^*P*<0.01 *versus* the t-BHP-treated group; ^e^*P*<0.05 *versus* RSV (10 *μ*M) and t-BHP (80 *μ*M) co-treated group with control siRNA transfection

The effects of RSV on regulation of mitochondrial redox status was evaluated using MitoSOX Red (Invitrogen, Carlsbad, CA, USA), a highly selective fluorescent probe for detection of the O_2_^•−^ generated within mitochondria. The MitoSOX Red reagent is live-cell permeant, and is selectively targeted to mitochondria. Once in mitochondria, MitoSOX Red is oxidized by O_2_^•−^ and exhibits red fluorescence.^19^ As shown in Figures 3a and b, t-BHP significantly increased mitochondrial O_2_^•−^ generation compared with the control group, which was notably reduced by RSV pretreatment (*P*<0.05). Moreover, this effect of RSV was sharply abolished by *Sirt3* siRNA transfection.

### RSV reduced mtROS generation by stimulating Sirt3-mediated mitochondrial enzyme activities and SOD2 deacetylation

**Figure 4:**
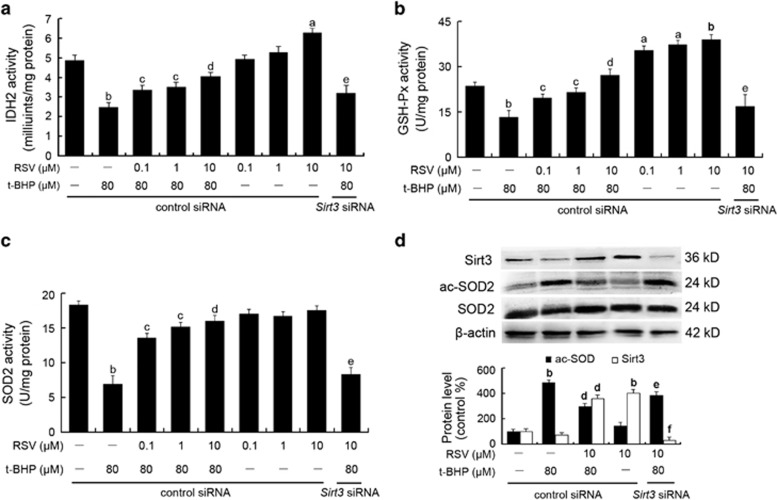
RSV reduced mtROS generation by stimulating Sirt3-mediated mitochondrial enzyme activities and SOD2 deacetylation. Cells were transfected with *Sirt3* siRNA as described in the Materials and Methods section. At 24-h post-transfection, cells were pretreated with different concentrations of RSV (0.1, 1 and 10 *μ*M) for 2 h and then treated with or without t-BHP of 80 *μ*M for an additional 4 h. (**a**–**c**) The enzyme activities of IDH2 (**a**), GSH-Px (**b**) and SOD2 (**c**) were determined using the corresponding assay kits, according to the manufacturer's instructions. (**d**) After the indicated treatments, the cells were harvested and lysed to detect protein levels of ac-SOD2 by western blot analysis. All results are presented as means±S.E.M. of at least three independent experiments. ^a^*P*<0.05, ^b^*P*<0.01 *versus* the control group; ^c^*P*<0.05, ^d^*P*<0.01 *versus* the t-BHP-treated group; ^e^*P*<0.05, ^f^*P*<0.01 *versus* RSV (10 *μ*M) and t-BHP (80 *μ*M) co-treated group with control siRNA transfection

The enzymatic activities of IDH2, SOD2 and GSH-Px that are involved in mtROS scavenging in endothelial cells were measured in this study. As shown in Figures 4a–c, t-BHP led to sharply decreased enzymatic activities of IDH2, SOD2 and GSH-Px, while RSV pretreatment significantly recovered these enzymatic activities (*P*<0.05). In addition, western blot analysis of mitochondrial extracts with an acetyl-SOD2 (ac-SOD2) antibody revealed that t-BHP increased the acetylation of SOD2, which was suppressed by RSV pretreatment (Figure 4d). However, the effects of RSV on radicals scavenging in HUVECs were significantly abolished by *Sirt3* siRNA transfection, indicating that Sirt3 plays a key role in the scavenging of excessive mtROS in HUVECs treated with RSV.

### RSV reduced mtROS generation through upregulation of Sirt3-mediated mtDNA transcription

**Figure 5:**
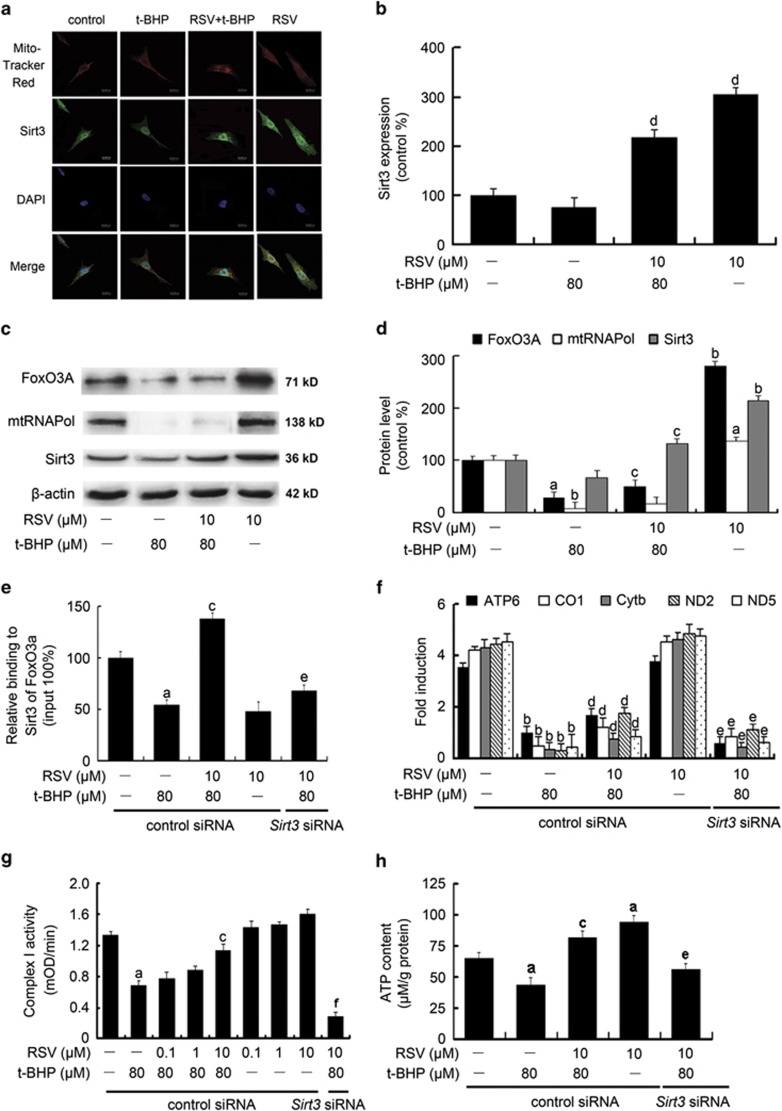
RSV reduced mtROS generation through the promotion of Sirt3-mediated mtDNA transcription. Cells were pretreated by RSV (10 *μ*M) for 2 h, then washed, and followed by treatment with or without t-BHP (80 *μ*M) for an additional 4 h. (**a**) Representative images show Sirt3 expression within mitochondria in HUVECs by immuofluorescence assay. Red fluorescence indicates mitochondria labeled with Mito-tracker Red. Green fluorescence originates from anti-Sirt3 labeling. And blue fluorescence indicates nuclei stained with DAPI. (**b**) The bar graph shows quantification of Sirt3 expression in the mitochondria. (**c**) The expressions of mtRNAPol, FoxO3A and Sirt3 in the mitochondria were determined by western blot. (**d**) The bar graph shows quantification of the indicated proteins. Sirt3 was knocked down by *Sirt3* siRNA transfection as described in the Materials and Methods section. The cells were pretreated with RSV of 10 *μ*M for 2 h, then washed, and followed by incubation with fresh medium in the presence or absence of t-BHP of 80 *μ*M for an additional 4 h. (**e**) Cells were harvested for ChIP analysis and the recruitment of FoxO3A was measured by real-time PCR assay using primers targeted to *Sirt3*. The results were plotted relative to those of the total input controls (untreated chromatin). (**f**) The expressions of mtDNA-encoded genes of ATP6, CO1, Cytb, ND2 and ND5 along with the housekeeping genes of *β*-actin, GAPDH and 18s rRNA were determined by real-time PCR assay. In order to generate a relative expression ratio, the expressions of the genes of interest were normalized to the geometric average of the housekeeping genes. (**g**) The enzyme activity of complex I was determined using the corresponding assay kit, according to the manufacturer's instructions. (**h**) ATP content was determined using an ATP determination kit. All results are presented as means±S.E.M. of at least three independent experiments. ^a^*P*<0.05, ^b^*P*<0.01 *versus* the control group; ^c^*P*<0.05, ^d^*P*<0.01 *versus* the t-BHP-treated group; ^e^*P*<0.05, ^f^*P*<0.01 *versus* RSV (10 *μ*M) and t-BHP (80 *μ*M) co-treated group with control siRNA transfection

Previous findings showed that FoxO3A co-precipitated with Sirt3 in the mitochondrial fractions of mammalian cells.^20^ Sirt3 has been shown to efficiently deacetylate FoxO3A, which is essential for the regulation of FoxO3A DNA-binding ability.^15^ In the current study, we found that RSV increased Sirt3 (green fluorescence by immunofluorescence assay) expression in mitochondria (red fluorescence with Mito-tracker Red) in HUVECs, supporting the hypothesis that RSV stimulates Sirt3 enrichment in mitochondria (Figures 5a and b). RSV addition also induced the expressions of mtRNA polymerase (mtRNAPol) and FoxO3A within mitochondria (Figures 5c and d). The chromatin immunoprecipitation (ChIP) analysis showed that FoxO3A and Sirt3 were co-recruited to mtDNA by RSV treatment, leading to the upregulation of mtDNA genes including ATP synthase 6 (ATP6), cytochrome *c* oxidase pseudogene 1 (CO1), cytochrome *b* (Cytb), NADH dehydrogenase 2 (ND2) and NADH dehydrogenase 5 (ND5; Figures 5e and f). Moreover, exposure to t-BHP resulted in a significant loss of complex I activity (Figure 5g) and decreased ATP synthesis (Figure 5h) in HUVECs (*P*<0.05), whereas pretreatment with RSV notably ameliorated t-BHP-induced decrease in mitochondrial ETC function. Furthermore, *Sirt3* siRNA transfection decreased the stimulatory effects of RSV on the mtDNA transcription and the coded gene expressions, along with the complex I activity and ATP content, which are possibly attributable to decreased deacetylation of FoxO3A mediated by Sirt3.^21^ These results clearly indicated that RSV pretreatment caused Sirt3 enrichment in mitochondria, which was required for the activation of mtDNA transcription and ATP synthesis, finally leading to a reduction of mtROS as the byproduct of OXPHOS in endothelial cells.

### RSV activated AMPK-PGC-1*α*-ERR*α*-Sirt3 signaling pathway in HUVECs

**Figure 6:**
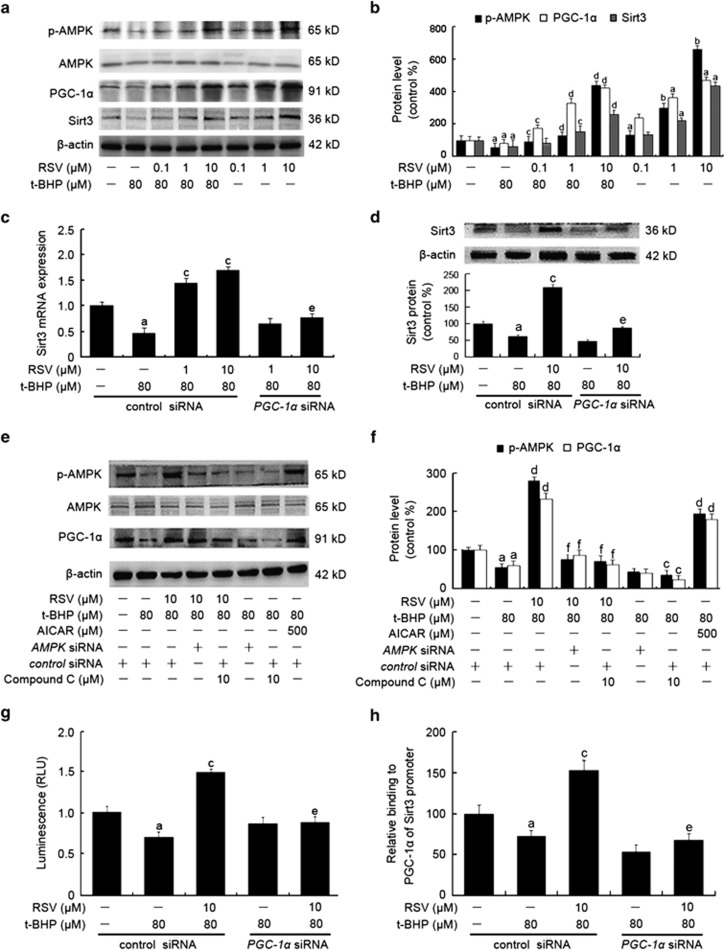
RSV stimulated AMPK-PGC-1*α*-ERR*α*-Sirt3 signaling pathway in HUVECs. (**a**) Cells were pretreated with RSV (0.1, 1 and 10 *μ*M) for 2 h and then treated with or without t-BHP of 80 *μ*M for an additional 4 h. The cells were harvested and lysed to detect protein levels of AMPK, p-AMPK, PGC-1*α* and Sirt3 by western blot assay. (**b**) The bar charts show quantification of the indicated proteins. Cells were transfected with *PGC-1α* siRNA as described in the Materials and Methods section. (**c**) The mRNA levels of the *Sirt3* genes were determined by real-time PCR assay. (**d**) The cells were collected and lysed, and Sirt3 protein levels were detected by western blot analysis. Cells were preincubated with compound C (10 *μ*M), *AMPK* siRNA or AICAR (500 *μ*M) as described in the Materials and Methods section. Then cells were pretreated with RSV of 10 *μ*M for 2 h, followed by incubation in the presence or absence of t-BHP of 80 *μ*M for an additional 4 h. (**e**) The expressions of AMPK, p-AMPK and PGC-1*α* were measured by western blot. (**f**) The bar charts show quantification of the indicated proteins. Cells were transfencted with *PGC-1α* siRNA as described in the Materials and Methods section. At 24-h post-transfection, the cells were pretreated with RSV of 10 *μ*M for 2 h and then treated with or without t-BHP of 80 *μ*M for an additional 4 h. (**g**) Cells were harvested using ChIP assay, and real-time PCR assay was carried out using primers specific to the *Sirt3* promoter. The results were determined relative to those of the total input controls (untreated chromatin). (**h**) Cells were co-transfected with pRL-TK reporter plasmid supplemented with ERRE-luc or NEG-PG04 (vehicle control), respectively. ERR*α* was knocked down by *ERRα* siRNA transfection, as described in the Materials and Methods section. And then cells were pretreated with RSV of 10 *μ*M for 2 h, followed by treatment with or without t-BHP of 80 *μ*M for an additional 4 h. Both firefly and Renilla luciferase activities were measured sequentially using the Dual-Glo luciferase reporter assay system. All results are presented as means±S.E.M. of at least three independent experiments. ^a^*P*<0.05, ^b^*P*<0.01 *versus* the control group; ^c^*P*<0.05, ^d^*P*<0.01 *versus* t-BHP-treated group; ^e^*P*<0.05, ^f^*P*<0.01 *versus* RSV (10 *μ*M) and t-BHP (80 *μ*M) co-treated group with control siRNA transfection

As shown in Figures 6a and b, treatment with RSV alone increased the protein expressions of p-AMPK, PGC-1*α* and Sirt3, which were in parallel with increased Sirt3 mRNA expression (Figure 6c). However, *PGC-1α* siRNA transfection prevented the induction of *Sirt3* mRNA and protein expression in HUVECs (Figures 6c and d), indicating that PGC-1*α* was required for the activation of RSV on Sirt3 expression. And the role of AMPK was further evaluated by the use of an AMPK inhibitor and agonist, as well as *AMPK* siRNA transfection. RSV-triggered AMPK activation was inhibited in the presence of compound C (a potent AMPK inhibitor) of 10 *μ*M or *AMPK* siRNA, accompanied by decreased expression of PGC-1*α* and Sirt3. In contrast, chemical activation of AMPK by AICAR (500 *μ*M) resulted in increased expression of phosphorylated AMPK (p-AMPK) and PGC-1*α* (Figures 6e and f). These results implicated that the AMPK-PGC-1*α*-Sirt3 axis was essential for the effect of RSV on mtROS homeostasis regulation in endothelial cells.

Furthermore, it has been demonstrated that estrogen-related receptor-*α* (ERR*α*) not only acts as a downstream target of PGC-1*α*, but is also co-activated by this transcriptional coactivator.^22^ Thus, we performed the luciferase assay to determine if Sirt3 mRNA activation by RSV occurred via PGC-1*α*-dependent ERR*α* binding to the Sirt3 promoter. We transfected HUVECs with an ERRE-luc reporter containing fragment of the Sirt3 promoter fused to a luciferase reporter gene. No firefly luciferase activities were measured in the vehicle group, suggesting that luciferase activities in cells co-transfected with ERRE-luc and pRL-TK vector were specific. As shown in Figure 6g, t-BHP exposure resulted in a decreased luciferase activity compared with the control group (*P*<0.05). In contrast, RSV pretreatment significantly increased ERRE-mediated Sirt3 transcriptional activity, whereas this effect was diminished by addition of *PGC-1α* siRNA. These results revealed that RSV activated Sirt3 mRNA transcription via a PGC-1*α*-dependent ERR*α*-mediated signaling pathway.

In addition, we performed a ChIP assay (Thermo Scientific, Waltham, MA, USA) to confirm weather RSV activated the binding of ERR*α* to the Sirt3 promoter. As shown in Figure 6h, t-BHP inhibited the amplification of a Sirt3 promoter fragment containing ERRE, while RSV pretreatment increased its amplification, an effect that was abolished by *ERRα* siRNA transfection. The findings clearly indicated that ERR*α* played an important role in mediating PGC-1*α*-induced Sirt3 expression. Overall, the AMPK-PGC-1*α*-ERR*α*-Sirt3 signaling pathway appears to be required for RSV-induced mtROS homeostasis and subsequent oxidative injury in endothelial cells.

## Discussion

**Figure 7:**
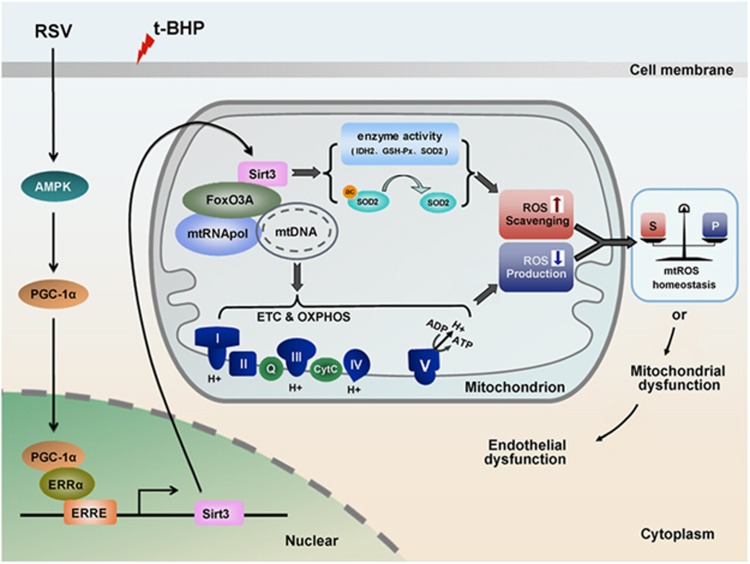
Proposed mechanism for RSV in the regulation of mtROS homeostasis through Sirt3 signaling pathway in HUVECs. RSV triggers AMPK-PGC-1*α* signaling pathway, which is required for ERR*α*-dependent Sirt3 transcription and subsequent enrichment within the mitochondria, thereby leading to deacetylation and activation of mitochondrial enzymes involved in mtROS regulation as well as stimulation of mitochondrial ETC efficiency by upregulation of mtDNA-encoded gene expression and ATP synthesis, finally contributing to mtROS homeostasis in HUVECs. I, complex I; II, complex II; III, complex III; IV, complex IV; V, ATP synthase enzymes; Q, CoQ; Cytc, cytochrome *c*

The results of this study, for the first time, support a model whereby RSV triggers AMPK-PGC-1*α* signaling, which is required for ERR*α*-dependent Sirt3 transcription and subsequent enrichment within the mitochondria. This in turn leads to deacetylation and activation of mitochondrial radical scavenging enzymes and mtDNA-encoded gene transcription and ATP synthesis, finally contributing to mtROS homeostasis in HUVECs (Figure 7). Our findings demonstrate a key role for Sirt3 in the inhibitory effects of RSV on the oxidative injury in endothelial cells, thus providing new insights into the cardiovascular benefits of RSV.

Increased oxidative stress plays an important role in the pathogenesis of cardiovascular diseases. The current study focused on the role of RSV in oxidative stress-induced endothelial cells. Oxidative stress is resulted from perturbation of cellular redox homeostasis, which is further associated with numerous deleterious consequences for the cell (e.g., lipid peroxidation or even cell death), and may play a critical role in endothelial dysfunction.^14^ In this study, t-BHP, a short chain analog of lipid hydroperoxides, was used as a pro-oxidant agent to induce oxidative stress in endothelial cells. Peroxy radicals can be generated from t-BHP in the cytosol by its interaction with ferrous iron in a reaction similar to the Fenton reaction, thereby leading to mitochondrial injury. The results suggested that t-BHP reduced cell viability and increased apoptosis in endothelial cells, accompanied by depolarized Δ*ψ*m and excessive O_2_^•−^ generation in the mitochondria, which were notably attenuated by RSV treatment. These findings revealed an RSV-mediated pharmacologic preconditioning effect in endothelial cells.

Increased ROS from vessel tissue under pathological conditions is known to be the main cause of endothelial dysfunction. Evidence suggests that mitochondria are the primary source of ROS, and homeostatic regulation of mtROS is influenced by various enzymes involved in the tricarboxylic acid (TCA), ETC, OXPHOS and free-radical-scavenging systems in the mitochondria.^23, 24^ The known site of mtROS production is the mitochondrial ETC, particularly complexes I and III, where O_2_^•−^ is formed and subsequently reduced by dismutation to H_2_O_2_. H_2_O can react with reduced Fe^2+^ and Cu^2+^ to produce highly toxic hydroxyl radicals (·OH) and can also penetrate membranes and leave the mitochondria. O_2_^•−^, H_2_O_2_ and ·OH are considered as the primary ROS. The mtROS scavenging system mainly includes SOD2, the glutathione and thioredoxin systems, which maintain a normal ratio of GSH/GSSG, NADPH/NADP^+^ and NADH/NAD^+^. In the current study, RSV treatment resulted in an increased enzymatic activities of IDH2, GSH-Px and SOD2, which were required for scavenging excessive mtROS in mitochondria. Moreover, MitoSOX Red, a highly selective fluorescent probe for O_2_^•−^ within mitochondria, was applied to detect mtROS and the ROS generated in the mitochondria could thus be differentiated from the cytoplasmic and exogenous sources.

Sirtuins are NAD^+^-dependent enzymes that have been implicated in a wide range of physiological and pathophysiological conditions through deacetylation of numerous substrates. Sirtuins are important regulators of mammalian physiology and have different levels of NAD^+^-dependent protein deacetylase activity.^25, 26^ Sirt3 has been shown to regulate mitochondrial metabolism by deacetylation of several enzymes involved in fatty acid oxidation, ketone body synthesis, TCA, OXPHOS, ROS generation and so on. Proteomics research has shown that several subunits of the enzymes in the mitochondrial ETC and TCA cycle have multiple acetylation modification sites, and increased acetylation was observed in Sirt3^−/−^ tissues, indicating that the enzyme activities may be regulated by Sirt3.^27, 28, 29^ In addition, mice lacking Sirt3 showed striking hyperacetylation of mitochondrial proteins, which were associated with accelerated development of metabolic syndrome.^30, 31, 32^ Our current findings suggested that RSV promotes SOD2 deacetylation in mitochondria, which was suppressed by *Sirt3* siRNA transfection, suggesting that Sirt3 plays a critical role in RSV-induced deacetylation in endothelial cells. SOD2 expression is believed to be an important cellular defense mechanism against to oxidative stress, whereas SOD2 deacetylation enhances mitochondrial scavenging capacity.^33, 34^

Recent studies have revealed that Sirt3 is involved in mtDNA transcription.^35^ Sirt3 mediates FoxO3A binding to mtDNA, and transcription of mitochondrial-encoded core or catalytic subunits of the OXPHOS machinery increases respiration, which in turn decreases the byproducts of mitochondrial electron transfer reactions from the incomplete reduction of oxygen. The decrease of mitochondrial ETC efficiency and ATP synthesis affects mtROS homeostasis, and alterations in mitochondrial physiology can increase the ATP/ROS ratio. A favorable balance in the ATP/ROS ratio may represent a common mechanism for extending the life span. Mitochondria in mammals undergoing caloric and dietary restrictions have been reported to show increased ATP/ROS ratios, in which cells generate the same amount of ATP with reduced oxidative stress and damage, with potential beneficial effects on life span.^36^ A similar positive effect on the ATP/ROS ratio was detected in adult flies, in which mitochondrial uncoupling in neurons was increased, and the life span extended.^37^ Interventions that maintain a beneficial mitochondrial ATP/ROS ratio could provide therapeutic opportunities for extending healthy life span and preventing disease.^38^ A previous study suggested that FoxO3A phosphorylation may be important for its mitochondrial import, and that once in the mitochondria, FoxO3A forms a complex with Sirt3 and mtRNAPol to activate transcription.^35^ As expected, RSV triggered the enrichment of Sirt3 in the mitochondria and activated the transcription of mtDNA-encoded OXPHOS units, which might be crucial for improving mitochondrial ETC efficiency (e.g., increased enzymatic activity of complex I) and reducing ROS generation, leading to sustained mtROS homeostasis. Our findings thus revealed a novel mechanism whereby RSV induction leads to an appropriate balance between mitochondrial energy production and mtROS generation, which is in turn responsible for the homeostatic regulation of redox status in HUVECs.

An expanding body of preclinical evidence suggests that RSV is associated with potential health benefits. Oral administration of RSV resulted in enhanced agonist-stimulated, endothelium-dependent relaxation in animal experiments^39^ and human trials;^40^ however, the exact molecular mechanisms remain unknown. To investigate the mechanism of RSV-induced Sirt3 activation, we examined the activation of AMPK and PGC-1*α*, sensors of energy molecules. AMPK, which is known to act as a fuel gauge and a master switch regulating glucose and lipid metabolism, senses metabolic stress and integrates diverse physiological signals to restore the energy balance.^41, 42^ It also serves as a regulator of cell survival or death in response to pathological stresses, such as hypoxia, osmotic stress and oxidative stress.^43^ AMPK thus plays a key role in intracellular metabolism and is an attractive therapeutic target, especially for energy-related diseases.^44^ In addition, fasting or nutrient excess may trigger the switching on/off of AMPK, which leads to alteration in PGC-1*α* activity.^45^ Furthermore, a previous report suggested that PGC-1*α* stimulated mouse Sirt3 activity in both muscle cells and hepatocytes,^46^ indicating that PGC-1*α* acts as an endogenous regulator of Sirt3. We therefore postulated that RSV regulates mtROS homeostasis through activation of the AMPK-PGC-1*α*-Sirt3 axis. As expected, our results demonstrated that RSV triggered AMPK phosphorylation and activation, which are required for PGC-1*α* expression. PGC-1*α* activated ERR*α* to cause ERR*α* coupling with ERRE in the Sirt3 promoter region, leading to Sirt3 mRNA transcription. These results are supported by those of Kong and colleagues.^46^ The AMPK-PGC-1/*α*-Sirt3 signaling pathway could be a key route in the regulation of mtROS homeostasis by RSV treatment in endothelial cells.

HUVECs were used in the study, though there might be differences between the endothelial cells from arterial and venous system.^47, 48, 49^ The first and most important difference between the two is the arteriovenous oxygen difference. As well, the endothelial cells in arteries and veins might be exposed to other different physical conditions (e.g., shear stress). However, the extent to which mitochondrial physiology varies in the endothelium of different tissues is unknown, although certainly some mitochondrial functions, such as mtDNA repair ability, appear to differ even among pulmonary arterial, venous and microvascular endothelial cells.^50^ On the opposite, both of the arterial and venous mitochondria in endothelial cells share similar characteristics. Particularly, the endothelial mitochondria from the arteries and veins are both proposed as the central oxygen sensors of local environments.^51^ Furthermore, from the perspective of developmental biology, it was found that arterial and venous endothelial cells have molecularly defined identities that are evident before circulatory flow or even tubulogenesis.^52^ Although AS only occurs in arteries but never in veins (mainly attributed to the higher blood pressure in arteries), isolation of vascular endothelial cells from veins (such as HUVECs) is commonly applied in several *in vitro* studies. Indeed, endothelial cells can also be harvested from arterier tissues, such as from the large vessels by mechanical removal or collagenase digestion,^53^ or from human internal mammary arteries obtained from donors undergoing coronary artery bypass graft surgery.^54^ However, direct study of vascular endothelial function in humans is also difficult in part because of limited access to arteries. Therefore, access to peripheral veins is more practical and the umbilical vein has been used due to its wide availability. To our knowledge, use of cells collected from veins to study the endothelial cells is a widespread way at present, even if the arterial-venous differences in endothelial cells might be present.

In conclusion, the results of this study provide novel insights into the interpretation of the potential mechanisms responsible for the homeostatic regulation of mtROS levels by RSV in endothelial cells, in which Sirt3 plays a key role. These results may provide valuable clues in the search for new drugs that can be applied to endothelial protection.

## Materials and Methods

### Chemicals and antibodies

Cell culture media HyQ M199/EBSS (M199) and fetal bovine serum were provided from HyClone Laboratories (Logan, UT, USA). RSV, t-BHP, dimethylsulfoxide (DMSO) and compound C were purchased from Sigma–Aldrich (St. Louis, MO, USA). The 5,5,‘6,6'-tetrachloro-1,1‘,3,3'-tetraethylbenzimidazolylcarbocyanine iodide (JC-1), 5-aminoimidazole-4-carboxamide 1-*β*-D-ribofuranoside (AICAR) and the antibody against *β*-actin were purchased from Beyotime Institute of Biotechnology (Beyotime Biotech, Beijing, China). A cell counting kit (CCK-8) was purchased from Dojindo Laboratories (Dojindo Molecular Technologies, Rockville, MD, USA). Antibodies against PGC-1*α*, Sirt3, Bcl-2, Bax, AIF and Cytc, mtRNAPol, FoxO3A, caspase 3, phosphorylated adenosine monophosphate-activated protein kinase (p-AMPK) and AMPK were obtained from Cell Signaling Technology (Beverly, MA, USA). MitoSOX Red mitochondrial superoxide indicator for live-cell imaging was obtained from Life Technologies (San Diego, CA, USA).

### Cell culture

HUVECs were isolated from umbilical veins from fresh cords obtained at normal deliveries as reported before.^55^ The study protocol was approved by the Ethics Review Committee of Third Military Medical University. The cells purity was identified >95% by immunofluorescence with specific anti-von Willebrand factor (vWF) antibody. HUVECs were cultured with M199 medium supplemented with 10% fetal bovine serum at 37 °C in a humidified atmosphere containing 5% CO_2_. All experiments were performed on the cells following 3–6 passages when the cells reached 80–90% confluence.

### Cell viability assay

Cell viability was measured by the CCK-8 assay according to the manufacturer's protocol. Briefly, cells were seeded in a 96-well microplate at a density of 8 × 10^3^ cells per well and then treated with different doses (20, 30, 40, 50, 60, 70, 80, 90 and 100 *μ*M) of t-BHP for 4 h. Meanwhile, cells were exposed to t-BHP of 80 *μ*M for different time intervals (1, 2, 4, 8, 12 and 24 h). In addition, cells were preincubated with RSV for 2 h at a series of concentrations (0.1, 0.5, 1, 5, 10 and 15 *μ*M, respectively). After removing the supernatant, cells were treated with or without t-BHP of 80 *μ*M for an additional 4 h. The control group was treated with 0.2% DMSO. Subsequently, CCK-8 solution (10 *μ*l/well) was added to each well and the plate was incubated at 37 °C for 2 h. The absorbance was measured using a monochromator microplate reader (Molecular Devices, Sunnyvale, CA, USA) at a wavelength of 450 nm. The percentage of living cells was calculated by the ratio of the optical density of the experimental cells to that of the normal cells (set as 100%).

### Flow cytometric analysis

Cell apoptosis was detected with annexin-V-fluorescein isothiocyanate (FITC) apoptosis detection kit (Beyotime Biotech, Beijing, China) as described by the manufacture's instructions. Briefly, cells were pretreated with different concentrations of RSV (0.1, 1 and 10 *μ*M) for 2 h, washed, and then treated with or without t-BHP (80 *μ*M) for another 4 h. Thereafter, cells were collected and washed with ice-cold PBS twice, gently resuspended in the binding buffer and incubated with annexin V-FITC and propidium iodide (PI) for 15 min in dark. Cells were analyzed on a FACScan flow cytometer (Becton Dickinson, Franklin Lakes, NJ, USA). The total apoptosis rate of cells was calculated as the sum of the rates of cells observed in the lower-right quadrant and the upper-right quadrant.

### Separation of cytosolic and mitochondrial fractions

The cytosolic and mitochondrial fractions were isolated as described.^56^ Briefly, 1 × 10^7^ cells were harvested by centrifugation at 800 × *g*, washed in PBS and re-pelleted. Cells were digitonin-permeablilized for 5 min on ice at a density of 3 × 10^7^/ml in cytosolic extraction buffer (75 mM NaCl, 1 mM NaH_2_PO_4_, 8 mM Na_2_HPO_4_, 250 mM sucrose, 1 mM phenylmethylsulfonyl fluoride (PMSF), 5 *μ*g/ml leupeptin, 5 *μ*g/ml aprotinin and 0.05% digitonin). Following the centrifugation step at 800 × *g* at 4 °C for 10 min, the supernatant was separated from the pellet comprising mitochondria and cellular debris. The supernatant containing cytoplasmic protein was further purified by centrifugation at 13 000 × *g* at 4 °C for 10 min. The precipitated mitochondrial fractions were collected and quantified for the protein content (3.5 mg/ml). And then, the pellets were solubilized in the same volume of mitochondrial lysis buffer (50 mM Tris (pH 7.4), 150 mM NaCl, 2 mM EDTA, 2 mM EGTA, 0.2% Triton X-100, 0.3% NP-40, 1 mM PMSF, 5 *μ*g/ml leupeptin and 5 *μ*g/ml aprotinin). After centrifugation at 12 000 × *g* at 4 °C for 10 min, supernatants were collected and used as the mitochondrial fraction, precipitation were collected and used as the cytoplasmic fraction.

### Western blotting

Cells were collected by scraping with ice-cold PBS containing PMSF, and were lysed in the buffer. The protein concentration of the lysates was determined by Bradford method. Protein (80 mg for each sample) was separated by sodium dodecyl sulfatepolyacrylamide gel (SDS-PAGE) and transferred to nitrocellulose membranes. After blocking (4% non-fat milk), the membranes were probed with the indicated primary antibody overnight at 4 °C, washed and incubated with a 1 : 6000 dilution of horseradish peroxidase (HRP)-conjugated secondary antibody for 1 h. The proteins were visualized by enhanced chemiluminescence (ECL; Amersham, UK) reagents for 1 min and exposed to CL-Xposure film (Pierce, Rockford, IL, USA). Finally, the blots were scanned, and densitometric analysis was performed on the scanning images using Scion Image-Release Beta 4.02 software (Scion Corporation, Frederick, MD, USA).

### RNA interference

The siRNAs for *AMPK* (human, sc-45312), *PGC-1α* (human, sc-38884), estrogen-related receptor *α* (*ERRα*; human, sc-44706) and *Sirt3* (human, sc-61555) along with control siRNA (human, sc-44230) and siRNA transfection reagent (human, sc-29528) were purchased from Santa Cruz Biotechnology (Santa Cruz, CA, USA). HUVECs were transfected with 100 nM siRNA for 5–7 h according to the manufacturer's protocol. Then, cells were switched into fresh complete medium and incubated for an additional 24 h. Thereafter, cells were harvested for further experiments.

### Mitochondrial membrane potential (Δ*ψ*m) determination

Mitochondrial membrane potential (Δ*ψ*m) was detected using the fluorescent, lipophilic dye, JC-1. JC-1 can easily penetrate cells with healthy mitochondria. A green fluorescent JC-1 probe exists as a monomer at low membrane potentials; however, at higher potentials, JC-1 forms red-fluorescent ‘J-aggregates'. The cells were incubated with 5 mg/l JC-1 for 1 h, washed twice with PBS and resuspended in the serum-free medium. The fluorescence intensity was determined by confocal laser scanning microscope (CLSM; model TCS SP2; Leica Microsystems GmbH, Wetzlar, Germany). For simultaneous visualization of the green fluorescence from JC-1 monomer and the red fluorescence from JC-1 J-aggregates, a long pass filter system was used (excitation lines 488 and 561 nm). Fixed exposure time and laser intensities were used during all acquisitions. The quantitative analysis of the red/green fluorescence signal was detected and the built-in evaluation software (Leica LAS AF Lite, Mannheim, Germany) was used for calculation. The ratio of green (JC-1 monomers) and red fluorescence (JC-1 J-aggregates) were measured within randomly selected cells. In both the experimental and control groups 30 individual cells were measured in six identical cultures. All data were statistically evaluated using the Mann–Whitney test and the Kolmogorov–Smirnov test. The Δ*ψ*m was represented as the ratio of red to green fluorescence intensity.

### *Mitochondrial O*_*2*_^•−^*assessment*

The mitochondrial O_2_^•−^ generation was assessed using the MitoSOX Red (Invitrogen), a highly selective fluorescent probe for the detection of O_2_^•−^ generated within mitochondria. HUVECs were added to glass coverslips at 8 × 10^3^ cells per well and were treated as indicated. MitoSOX Red reagent stock solution was diluted with HBSS/Ca/Mg buffer to make a working solution of 5 *μ*M. Then, cells were incubated with 5 *μ*M MitoSOX Red reagent in dark at 37 °C for 10 min. Cells were washed gently three times with warm buffer and were stained with counterstains as desired amount for imaging. Fluorescence intensities of mtROS were determined using an Infinite M200 microplate reader (Tecan Group Ltd., Männedorf, Switzerland) and images were visualized by a CLSM (model TCS SP2; Leica Microsystems GmbH) with the emission wavelength of 579 nm and the excitation wavelength of 510 nm, respectively.

### Mitochondrial enzyme activities

The enzyme activities of complex I, IDH2, SOD2 and GSH-Px were assessed in mitochondrial homogenates, which contained protease inhibitors only. The complex I activity was measured with complex I enzyme activity microplate assay kit (Abcam, Cambridge, UK) and its activity was determined from the oxidation of reduced nicotinamide adenine dinucleotide (NADH/NAD^+^) to NAD^+^. The IDH2 activity was assayed by IDH activity assay kit (Sigma–Aldrich) with a temperature-controlled spectrofluorometer (Hitachi F-4500, Tokyo, Japan) at the excitation wavelength of 366 nm and emission wavelength of 450 nm. And the activities of SOD2 and GSH-Px in the mitochondria were determined by commercial assay kits purchased from Nanjing Jiancheng Bioengineering Institute (Nanjing, Jiangsu, China), according to the manufacturer's instructions, respectively.

### Immunoprecipitation of acetyl lysine-modified SOD2

Mitochondrial proteins (150 *μ*g) were incubated at 4 °C with 5 *μ*g of antibody against acetyl lysine (K68) SOD2 (ac-SOD2; Cambridge, MA, USA) in a total volume of 250 *μ*l for 24 h. Following addition of protein A/G-agarose (50 *μ*l; Santa Cruz Biotechnology) the solution was incubated for 2 h at 4 °C, centrifuged and the agarose complexes extensively washed with Tris-buffered saline containing 0.05% (v/v) Tween 20 (Sigma–Aldrich). Pellets were stored at −20 °C until further analysis by SDS-PAGE.

### Immunofluorescence imaging

Confluent HUVECs were grown on glass coverslips and then harvested. The slides were incubated with the Mito-Tracker Red (Invitrogen) of 80 nM for 20 min, and then were fixed with 4% (v/v) paraformaldehyde for 20 min at 37 °C and permeabilized with 0.1% Triton X-100 for 10 min at room temperature. The slides were blocked with PBS supplemented with 0.1% (w/v) BSA and 0.1% (w/v) Tween 20, thereafter, incubated with anti-Sirt3 antibody (1 : 50 dilution) overnight at 4 °C. After washing with PBS, the slides were incubated for 1 h at room temperature with a FITC-conjugated secondary antibody (1 : 1000 dilution; Molecular Probes, Eugene, OR, USA). Finally, the slides were washed twice with PBS and the nuclei were counterstained with 4′, 6′-diamidino-2-phenylindole (DAPI) for 5 min at room temperature. Images of the stained cells were obtained using CLSM (model TCS SP2; Leica Microsystems GmbH) and the intracellular fluorescence intensity was expressed as the percentage relative to the control group (set as 100%), respectively.

### Real-time PCR

Total cellular RNA was extracted by Trizol RNA isolation kit (Invitrogen) according to the manufacture's protocol. Then, cDNA was synthesized from 1 *μ*g RNA for each sample with random primers according to the protocol of Invitrogen. Quantitative real-time PCR was performed on the iQ5 multicolor real-time PCR detection system (Bio-Rad, Hertfordshire, UK) with specific primers (Table 1). All primers were designed by the software Primer Premier 5.0 and synthesized by Invitrogen (Shanghai, China). In the reaction, 1 *μ*l cDNA of each sample was mixed with SYBR Green JumpStart Taq ReadyMix (Fisher Scientific, Atlanta, GA, USA) according to the manufacture's instructions. And the PCR conditions: denaturation step of 5 min at 95 °C; amplification for 40 cycles (denaturation: 30 s at 95 °C; annealing: 30 s at corresponding temperature; extension: 30 s at 72°C); final extension for 7 min at 72°C. All the samples have three parallel wells and the experiments were repeated at least three times. The melt-curve analysis was used to confirm specific product formation. Housekeeping genes used in this study were *β*-actin, glyceraldehyde-3-phosphate dehydrogenase (GAPDH) and 18s ribosomal RNA (18s rRNA). In order to generate a relative expression ratio, the mRNA level of the gene of interest was normalized to the geometric average mRNA level of the housekeeping genes.

### Chromatin immunoprecipitation analysis of genomic and mtDNA

ChIP analysis for genomic DNA and mtDNA was performed with Pierce Agarose ChIP kit as described previously with minor modifications.^57^ Briefly, formaldehyde was added directly to tissue culture medium to a final concentration of 1%. Cross-linking was allowed to proceed for 15 min at room temperature and then stopped by the addition of glycine to a final concentration of 0.125 M. Cross-linked cells were scraped, washed with PBS, and then lysed in CLB (10 mM Tris (pH 8.0), 10 mM NaCl and 0.2% NP-40) with protease inhibitors (1 mM phenylmethylsulphonylfluoride, 5 mM NaF, 1 mM Na_3_VO_4_, 1,5 *μ*M pepstatin, 2 *μ*M leupeptin, 10 *μ*g/ml aprotinin, 1 mM NaB and 40 *μ*M bestatin). The mitochondrial fraction was enriched and lysed by mitochondrial lysis buffer (50 mM Tris–Cl pH 8.1, 10 mM EDTA and 1% SDS) or purified using the Qproteome mitochondria isolation kit (Qiagen, Valencia, CA, USA). The chromatin solution was sonicated, cleared by centrifugation and the supernatant was collected; 1% of the supernatant was taken as input. Five micrograms of antibody per point was used to immunoprecipitate chromatin-bound complexes. Immunoprecipitation was performed on a rotating platform overnight at 4 °C. Antibodies against PGC-1*α* (for genomic DNA) and Sirt3 (for mtDNA) were used for ChIP analysis, respectively. And the monoclonal IgG was used as unrelated antibodies. Immunocomplexes were pulled down using protein G (GE Healthcare, Milwaukee, WI, USA). Following extensive washing, bound DNA was reverse cross-linked and purified, then analyzed by real-time PCR assay as described previously.^44^ The primers for Sirt3 promotor was 5′-AGTAAGACCCGAGGTAGGAG-3′ (forward) and 5′-GGAATAAGAGGGATGACAGA-3′ (reverse).

### Adenosine triphosphate quantification

HUVECs were grown on glass coverslips at a density of 1.0 × 10^6^ cells per well and then harvested. Cells were treated as indicated, then ATP content was measured using an ATP determination kit (Invitrogen, Carlsbad, CA, USA) according to the manufacturer's instructions. Briefly, the cells were washed twice in PBS and lysed with 500 *μ*l of 0.2 M HCLO_4_ and centrifuged at 5000 × *g* for 10 min. The supernatant was mixed with the ATP determination kit reaction buffer containing luciferin and luciferase. After gentle mixing, the readings for the above mixtures were taken with the luminometer (Turner Designs, Sunnyvale, CA, USA). ATP contents were calculated by using an ATP standard curve.

### Luciferase assay

HUVECs (1 × 10^5^ cells per well) were maintained in 24-well plates until 60–70% confluence. Transient transfection was preformed by Lipofectamine 2000 (Invitrogen) transfection reagent according to the manufacturer's protocol. Briefly, cells were co-transfected with 0.2 *μ*g/well of estrogen receptor response element (ERRE)-luc plasmid HPRM24033-PG04 (Promega, Madison, WI, USA; Supplementary Figure 1A) and pRL-TK reporter plasmid (Promega; Supplementary Figure 1B), respectively. The mass of transfected plasmids was balanced with empty vector NEG-PG04 (Promega; Supplementary Figure 1C). After transfection for 48 h, cells were treated with RSV (10 *μ*M) for 2 h and followed by t-BHP (80 *μ*M) treatment for an additional 4 h. Thereafter, cells were lysed and both of the firefly and Renilla luciferase activities were measured sequentially with the Dual-Glo luciferase reporter assay system (Promega) according to the manufacturer's instructions.

### Statistical analysis

All the experiments were performed at least three times and results are expressed as the mean±S.E.M. Raw data were analyzed with SPSS 13.0 software (Chicago, IL, USA). Statistical analysis for multiple group comparisons was performed by one-way analysis of variance, followed by *post hoc* least significant difference tests. A two-sided *P*-value <0.05 was considered statistically significant.

## Figures and Tables

**Table 1:** Primer details

**Primer**	**Forward (5′-3′)**	**Reverse (5′-3′)**	**Annealing temperatures (°C)**
Atp 6	GCCGCAGTACTGATCATTCTATTTC'	TCGGTTGTTGATGAGATATTTGGA	55
COX 1	CCCCGCATAAACAACATAAGC	CAGATGCGAGCAGGAGTAGGA	57
ND2	AAACCCTCGTTCCACAGAAGC'	GGATTATGGATGCGGTTGCT	58
ND5	GCAGCCATTCAAGCAATCCTA	AGGCGAGGATGAAACCGATA	60
Cyto b	TCGGCATTATCCTCCTGCTT	CTCACGGGAGGACATAGCCTCT	51
Sirt3	CCCAGTGGCATTCCAGACTT	AAGGGCTTGGGGTTGTGAAA	55
GAPDH	CAACTTTGGTATCGTGGAAGGAC	ACAGTCTTCTGGGTGGCAGTG	60
*β*-actin	CTGGCACCCAGCACAATG	GTTCATGAGGCACACCTAGCC	55
18s rRNA	GAGTATGGTTGCAAAGCTGAAACTT	TGAGTCAAATTAAGCCGCAGG	58

## Abbreviations

ACO: aconitase
AIF: apoptosis-inducing factor
AMPK: adenosine monophosphate-activated protein kinase
AS: atherosclerosis
ATP: adenosine triphosphate
ATP6: ATP synthase 6
Bax: Bcl-2-associated X protein
Bcl-2: B-cell lymphoma-2
BSA: bovine serum albumin
CAD: cardiovascular disease
CCK-8: cell counting kit
cDNA: complementary DNA
ChIP: Chromatin immunoprecipitation
CO1: cytochrome *c* oxidase pseudogene 1
Cytb: cytochrome *b*
Cytc: Cytochrome *c*
DAPI: 4′, 6′-diamidino-2-phenylindole
DMSO: dimethylsulfoxide
ETC: electron transport chain
ETFDH: electron transfer flavoprotein dehydrogenase
ERR*α*: estrogen-related receptor *α*
ERRE: estrogen receptor response element
FITC: fluorescein isothiocyanate
FoxO3A: Forkhead box O3A
GAPDH: glyceraldehyde-3-phosphate dehydrogenase
GPD2: glycerol phosphate dehydrogenase
GSSG: oxidized glutathione
GSH-Px: glutathione peroxidase
HUVECs: human umbilical vein endothelial cells
IDH2: isocitrate dehydrogenase 2
JC-1: 5,5,‘6,6'-tetrachloro-1,1‘,3,3'-tetraethylbenzimidazolylcarbocyanine iodide
KGDH: *α*-keto glutaric dehydrogenase
mtROS: mitochondrial reactive oxygen species
NAD^+^: nicotinamide adenine nucleotide, oxidized form
NADPH: nicotinamide adenine dinucleotide, reduced form
ND2: NADH dehydrogenase 2
ND5: NADH dehydrogenase 5
OXPHOS: oxidative phosphorylation
PDH: pyruvate dehydrogenase
PGC-1*α*: peroxisome proliferator-activated receptor-*γ* coactivator-1 *α*
PI: propidium iodide
ROS: reactive oxygen species
RSV: resveratrol
SDS-PAGE: sodium dodecyl sulfatepolyacrylamide gel
siRNA: small-interfering RNA
Sirt3: silent mating-type information regulation 2 homolog 3
SOD2: manganese superoxide dismutase
t-BHP: tert-butyl hydroperoxide
TCA: tricarboxylic acid
Δ*ψ*m: mitochondrial membrane potential
