# Supplementary figures and images for: Resveratrol regulates mitochondrial reactive oxygen species homeostasis through Sirt3 signaling pathway in human vascular endothelial cells
Source: Cell Death Dis. 2014 Dec 18;5(12):e1576–. doi: 10.1038/cddis.2014.530 (PMC4454164; doi:10.1038/cddis.2014.530)

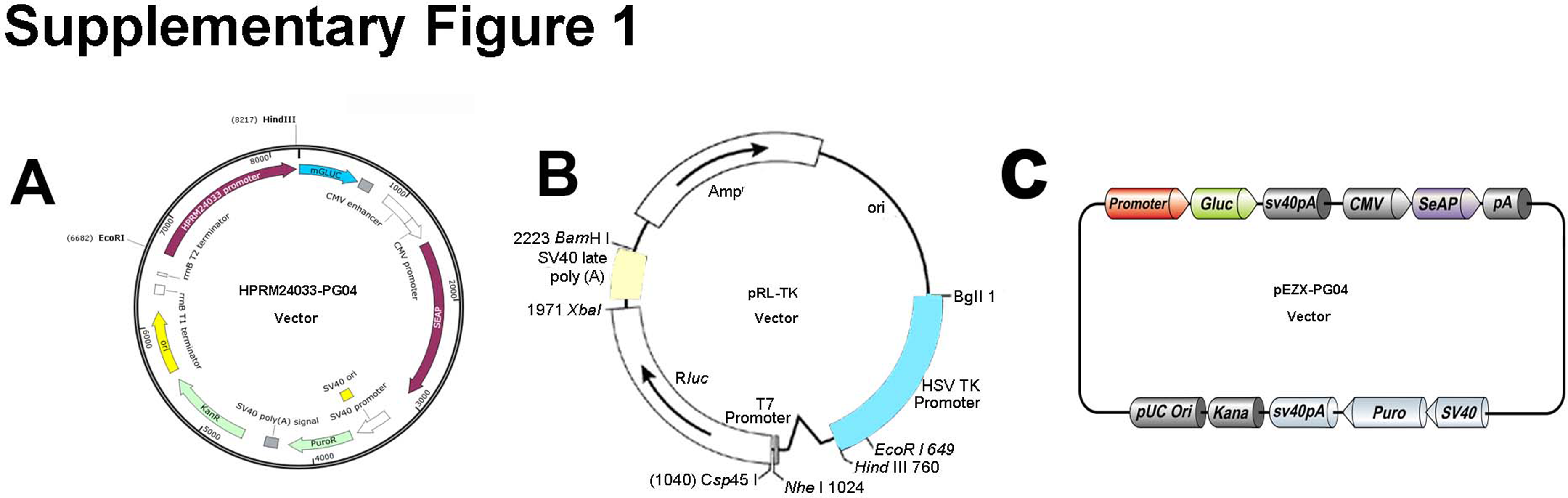

Supplement: Supplementary Figure 1 [file cddis2014530x1.tif]
